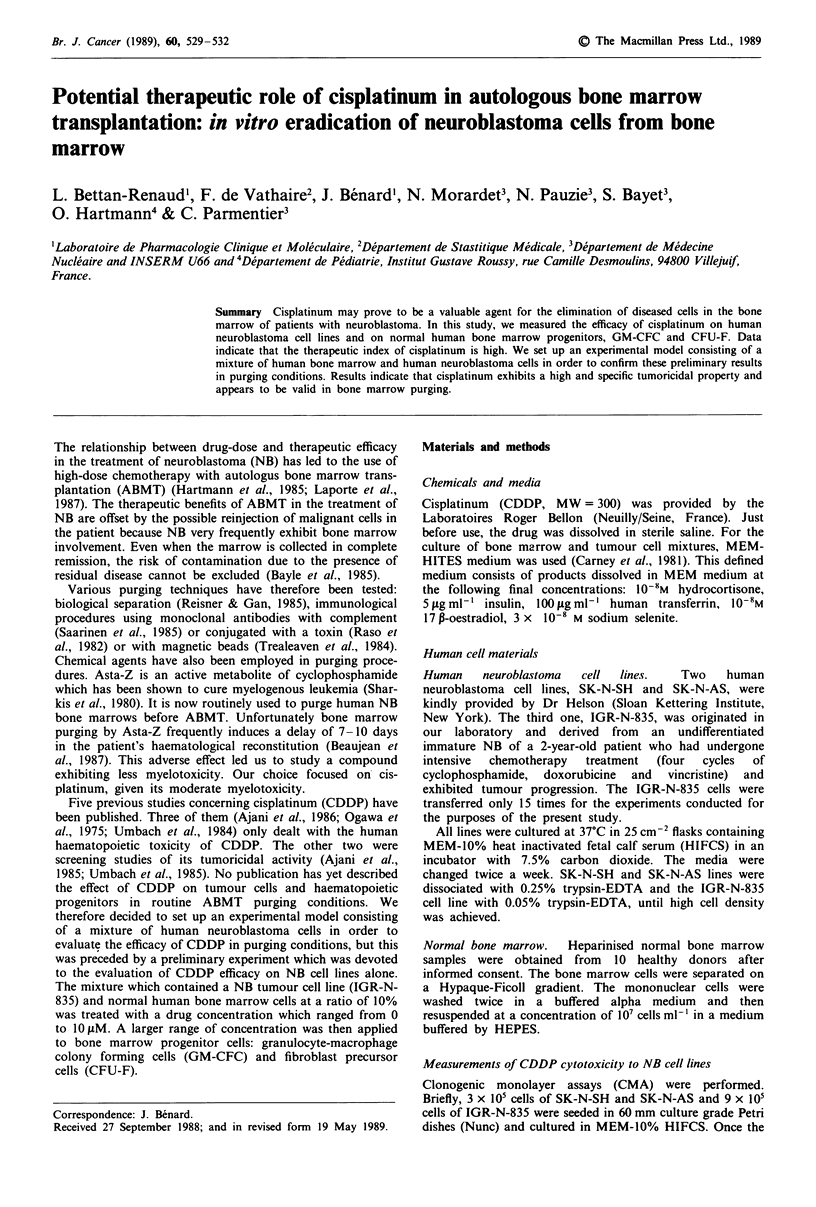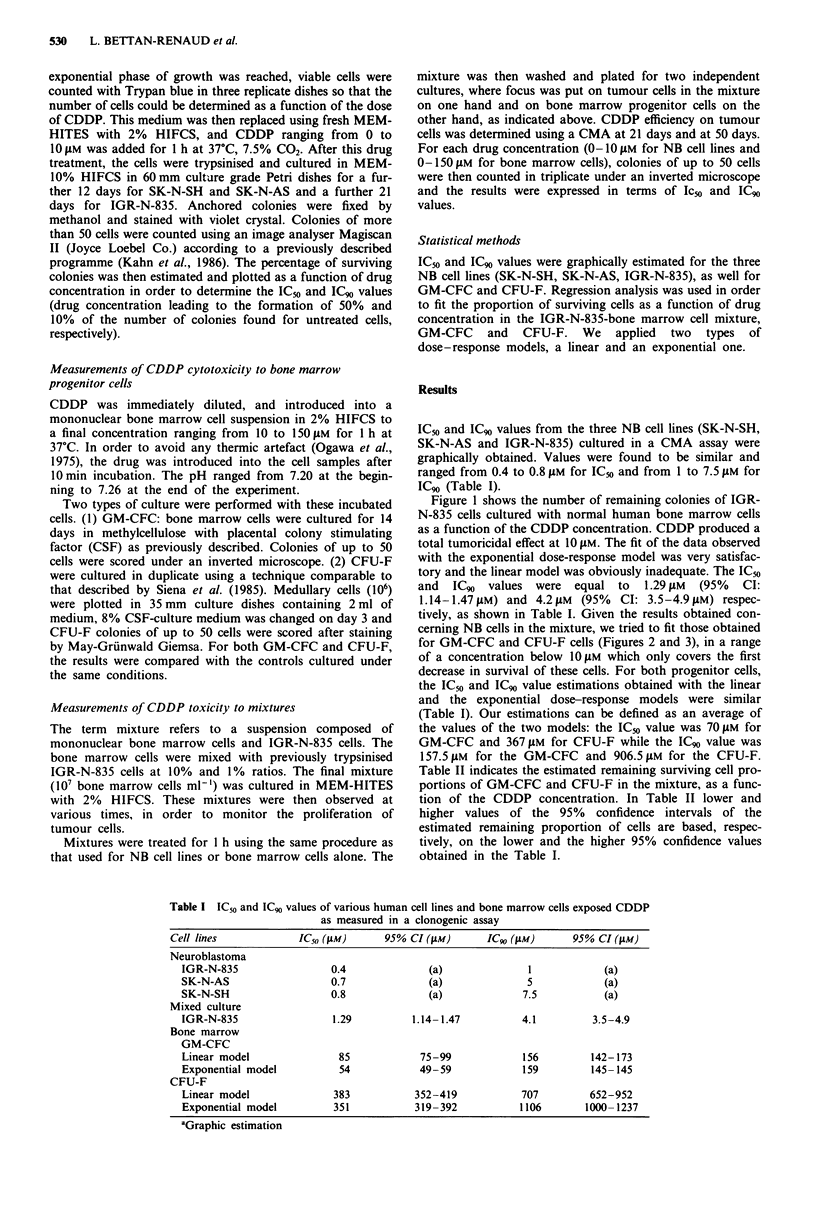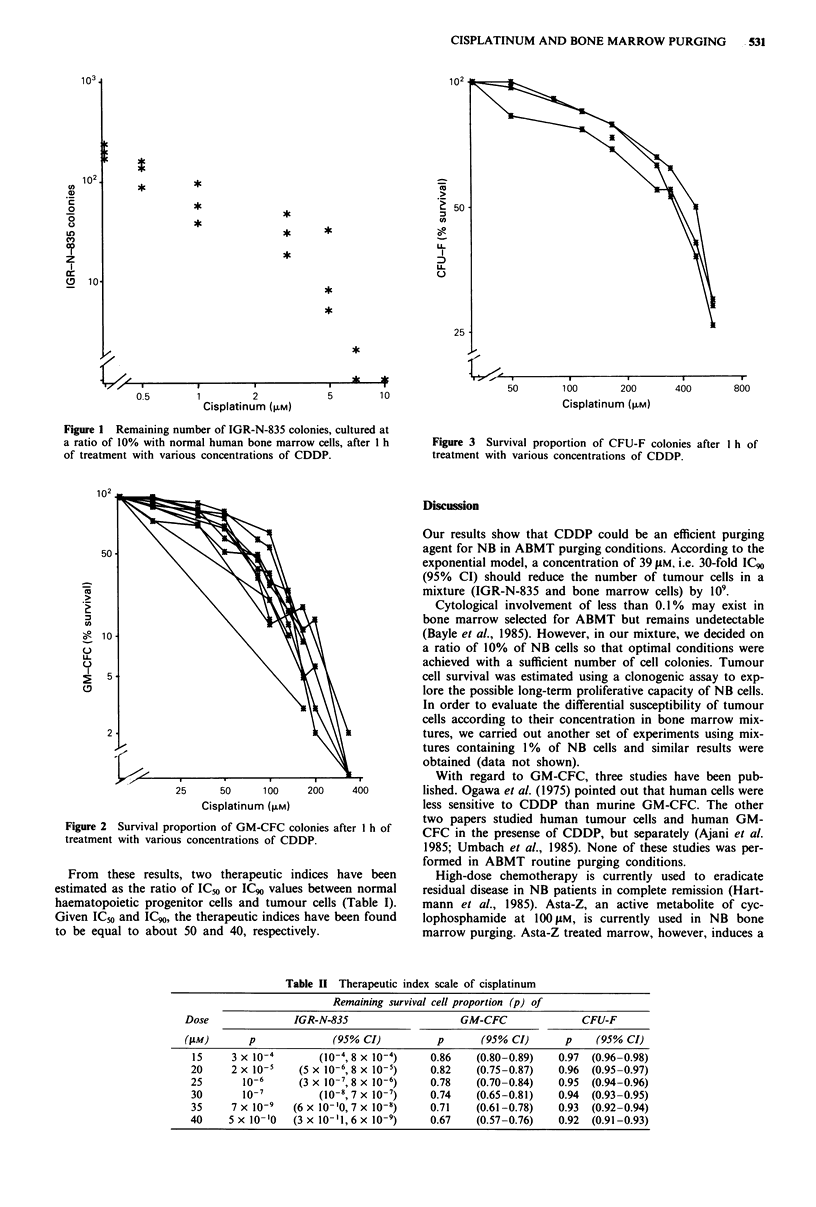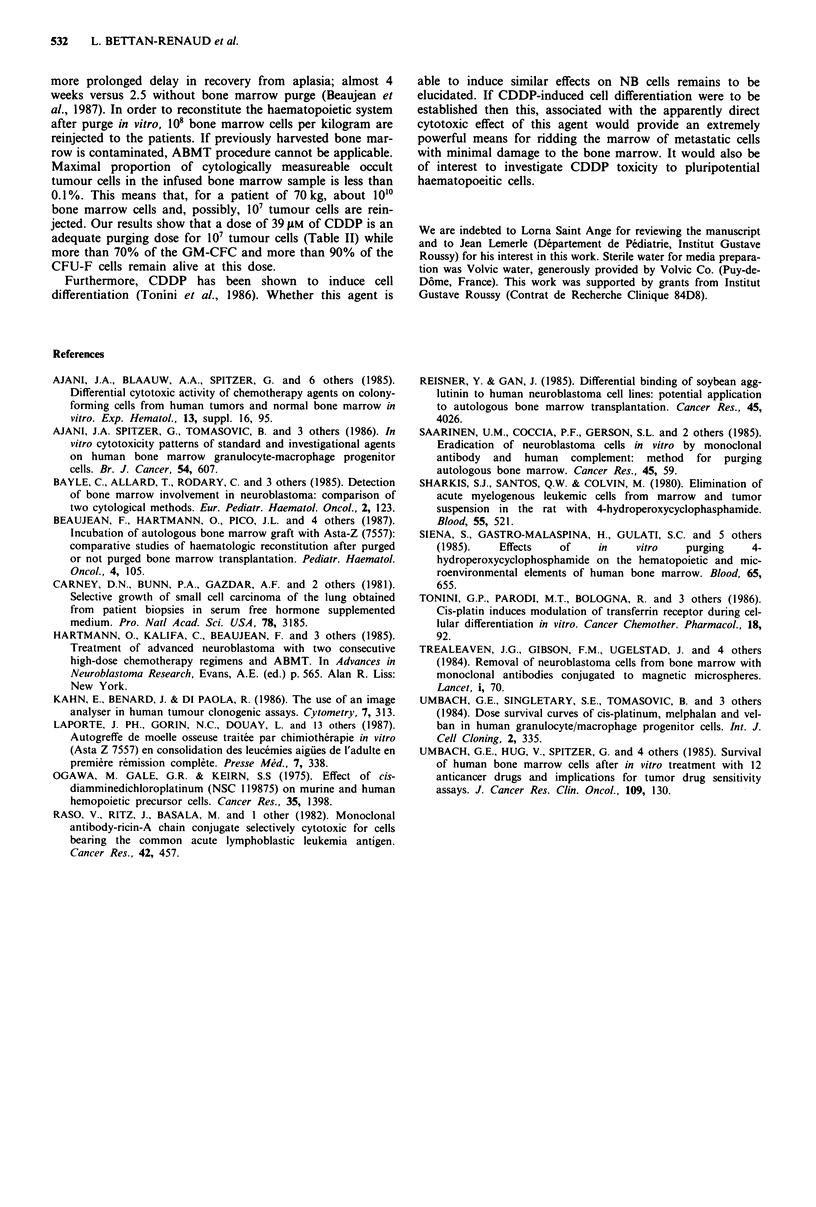# Potential therapeutic role of cisplatinum in autologous bone marrow transplantation: in vitro eradication of neuroblastoma cells from bone marrow.

**DOI:** 10.1038/bjc.1989.307

**Published:** 1989-10

**Authors:** L. Bettan-Renaud, F. de Vathaire, J. BÃ©nard, N. Morardet, N. Pauzie, S. Bayet, O. Hartmann, C. Parmentier

**Affiliations:** Laboratoire de Pharmacologie Clinique et MolÃ©culaire, Institut Gustave Roussy, Villejuif, France.

## Abstract

Cisplatinum may prove to be a valuable agent for the elimination of diseased cells in the bone marrow of patients with neuroblastoma. In this study, we measured the efficacy of cisplatinum on human neuroblastoma cell lines and on normal human bone marrow progenitors, GM-CFC and CFU-F. Data indicate that the therapeutic index of cisplatinum is high. We set up an experimental model consisting of a mixture of human bone marrow and human neuroblastoma cells in order to confirm these preliminary results in purging conditions. Results indicate that cisplatinum exhibits a high and specific tumoricidal property and appears to be valid in bone marrow purging.


Br. J. Cancer (1989), 60, 529–532 © The Macmillan Press Ltd., 1989

### Potential therapeutic role of cisplatinum in autologous bone marrow transplantation: *in vitro* eradication of neuroblastoma cells from bone marrow

L. Bettan-Renaud[1], F. de Vathaire[2], J. Bénard[1], N. Morardet[3], N. Pauzie[3], S. Bayet[3], O. Hartmann[4] & C. Parmentier[3]

[1]*Laboratoire de Pharmacologie Clinique et Moléculaire,* [2]*Département de Stastitique Médicale,* [3]*Département de Médecine Nucléaire and INSERM U66 and* [4]*Département de Pédiatrie, Institut Gustave Roussy, rue Camille Desmoulins, 94800 Villejuif, France.*

**Summary** Cisplatinum may prove to be a valuable agent for the elimination of diseased cells in the bone marrow of patients with neuroblastoma. In this study, we measured the efficacy of cisplatinum on human neuroblastoma cell lines and on normal human bone marrow progenitors, GM-CFC and CFU-F. Data indicate that the therapeutic index of cisplatinum is high. We set up an experimental model consisting of a mixture of human bone marrow and human neuroblastoma cells in order to confirm these preliminary results in purging conditions. Results indicate that cisplatinum exhibits a high and specific tumoricidal property and appears to be valid in bone marrow purging.

The relationship between drug-dose and therapeutic efficacy in the treatment of neuroblastoma (NB) has led to the use of high-dose chemotherapy with autologus bone marrow transplantation (ABMT) (Hartmann *et al.*, 1985; Laporte *et al.*, 1987). The therapeutic benefits of ABMT in the treatment of NB are offset by the possible reinjection of malignant cells in the patient because NB very frequently exhibit bone marrow involvement. Even when the marrow is collected in complete remission, the risk of contamination due to the presence of residual disease cannot be excluded (Bayle *et al.*, 1985).

Various purging techniques have therefore been tested: biological separation (Reisner & Gan, 1985), immunological procedures using monoclonal antibodies with complement (Saarinen *et al.*, 1985) or conjugated with a toxin (Raso *et al.*, 1982) or with magnetic beads (Trealeaven *et al.*, 1984). Chemical agents have also been employed in purging procedures. Asta-Z is an active metabolite of cyclophosphamide which has been shown to cure myelogenous leukemia (Sharkis *et al.*, 1980). It is now routinely used to purge human NB bone marrows before ABMT. Unfortunately bone marrow purging by Asta-Z frequently induces a delay of 7–10 days in the patient's haematological reconstitution (Beaujean *et al.*, 1987). This adverse effect led us to study a compound exhibiting less myelotoxicity. Our choice focused on cisplatinum, given its moderate myelotoxicity.

Five previous studies concerning cisplatinum (CDDP) have been published. Three of them (Ajani *et al.*, 1986; Ogawa *et al.*, 1975; Umbach *et al.*, 1984) only dealt with the human haematopoietic toxicity of CDDP. The other two were screening studies of its tumoricidal activity (Ajani *et al.*, 1985; Umbach *et al.*, 1985). No publication has yet described the effect of CDDP on tumour cells and haematopoietic progenitors in routine ABMT purging conditions. We therefore decided to set up an experimental model consisting of a mixture of human neuroblastoma cells in order to evaluate the efficacy of CDDP in purging conditions, but this was preceded by a preliminary experiment which was devoted to the evaluation of CDDP efficacy on NB cell lines alone. The mixture which contained a NB tumour cell line (IGR-N-835) and normal human bone marrow cells at a ratio of 10% was treated with a drug concentration which ranged from 0 to 10 μM. A larger range of concentration was then applied to bone marrow progenitor cells: granulocyte-macrophage colony forming cells (GM-CFC) and fibroblast precursor cells (CFU-F).

Correspondence: J. Bénard.
Received 27 September 1988; and in revised form 19 May 1989.

### Materials and methods

### *Chemicals and media*

Cisplatinum (CDDP, MW = 300) was provided by the Laboratoires Roger Bellon (Neuilly/Seine, France). Just before use, the drug was dissolved in sterile saline. For the culture of bone marrow and tumour cell mixtures, MEM-HITES medium was used (Carney *et al.*, 1981). This defined medium consists of products dissolved in MEM medium at the following final concentrations: $10^{-8}$M hydrocortisone, 5 $\mu g\,ml^{-1}$ insulin, 100 $\mu g\,ml^{-1}$ human transferrin, $10^{-8}$M 17 β-oestradiol, $3 \times 10^{-8}$ M sodium selenite.

### *Human cell materials*

*Human neuroblastoma cell lines.* Two human neuroblastoma cell lines, SK-N-SH and SK-N-AS, were kindly provided by Dr Helson (Sloan Kettering Institute, New York). The third one, IGR-N-835, was originated in our laboratory and derived from an undifferentiated immature NB of a 2-year-old patient who had undergone intensive chemotherapy treatment (four cycles of cyclophosphamide, doxorubicine and vincristine) and exhibited tumour progression. The IGR-N-835 cells were transferred only 15 times for the experiments conducted for the purposes of the present study.

All lines were cultured at 37°C in 25 $cm^{-2}$ flasks containing MEM-10% heat inactivated fetal calf serum (HIFCS) in an incubator with 7.5% carbon dioxide. The media were changed twice a week. SK-N-SH and SK-N-AS lines were dissociated with 0.25% trypsin-EDTA and the IGR-N-835 cell line with 0.05% trypsin-EDTA, until high cell density was achieved.

*Normal bone marrow.* Heparinised normal bone marrow samples were obtained from 10 healthy donors after informed consent. The bone marrow cells were separated on a Hypaque-Ficoll gradient. The mononuclear cells were washed twice in a buffered alpha medium and then resuspended at a concentration of $10^7$ cells $ml^{-1}$ in a medium buffered by HEPES.

### *Measurements of CDDP cytotoxicity to NB cell lines*

Clonogenic monolayer assays (CMA) were performed. Briefly, $3 \times 10^5$ cells of SK-N-SH and SK-N-AS and $9 \times 10^5$ cells of IGR-N-835 were seeded in 60 mm culture grade Petri dishes (Nunc) and cultured in MEM-10% HIFCS. Once the exponential phase of growth was reached, viable cells were counted with Trypan blue in three replicate dishes so that the number of cells could be determined as a function of the dose of CDDP. This medium was then replaced using fresh MEM-HITES with 2% HIFCS, and CDDP ranging from 0 to 10 μM was added for 1 h at 37°C, 7.5% $CO_2$. After this drug treatment, the cells were trypsinised and cultured in MEM-10% HIFCS in 60 mm culture grade Petri dishes for a further 12 days for SK-N-SH and SK-N-AS and a further 21 days for IGR-N-835. Anchored colonies were fixed by methanol and stained with violet crystal. Colonies of more than 50 cells were counted using an image analyser Magiscan II (Joyce Loebel Co.) according to a previously described programme (Kahn *et al.*, 1986). The percentage of surviving colonies was then estimated and plotted as a function of drug concentration in order to determine the $IC_{50}$ and $IC_{90}$ values (drug concentration leading to the formation of 50% and 10% of the number of colonies found for untreated cells, respectively).

*Measurements of CDDP cytotoxicity to bone marrow progenitor cells*

CDDP was immediately diluted, and introduced into a mononuclear bone marrow cell suspension in 2% HIFCS to a final concentration ranging from 10 to 150 μM for 1 h at 37°C. In order to avoid any thermic artefact (Ogawa *et al.*, 1975), the drug was introduced into the cell samples after 10 min incubation. The pH ranged from 7.20 at the beginning to 7.26 at the end of the experiment.

Two types of culture were performed with these incubated cells. (1) GM-CFC: bone marrow cells were cultured for 14 days in methylcellulose with placental colony stimulating factor (CSF) as previously described. Colonies of up to 50 cells were scored under an inverted microscope. (2) CFU-F were cultured in duplicate using a technique comparable to that described by Siena *et al.* (1985). Medullary cells ($10^6$) were plotted in 35 mm culture dishes containing 2 ml of medium, 8% CSF-culture medium was changed on day 3 and CFU-F colonies of up to 50 cells were scored after staining by May-Grünwald Giemsa. For both GM-CFC and CFU-F, the results were compared with the controls cultured under the same conditions.

*Measurements of CDDP toxicity to mixtures*

The term mixture refers to a suspension composed of mononuclear bone marrow cells and IGR-N-835 cells. The bone marrow cells were mixed with previously trypsinised IGR-N-835 cells at 10% and 1% ratios. The final mixture ($10^7$ bone marrow cells $ml^{-1}$) was cultured in MEM-HITES with 2% HIFCS. These mixtures were then observed at various times, in order to monitor the proliferation of tumour cells.

Mixtures were treated for 1 h using the same procedure as that used for NB cell lines or bone marrow cells alone. The mixture was then washed and plated for two independent cultures, where focus was put on tumour cells in the mixture on one hand and on bone marrow progenitor cells on the other hand, as indicated above. CDDP efficiency on tumour cells was determined using a CMA at 21 days and at 50 days. For each drug concentration (0–10 μM for NB cell lines and 0–150 μM for bone marrow cells), colonies of up to 50 cells were then counted in triplicate under an inverted microscope and the results were expressed in terms of $Ic_{50}$ and $IC_{90}$ values.

*Statistical methods*

$IC_{50}$ and $IC_{90}$ values were graphically estimated for the three NB cell lines (SK-N-SH, SK-N-AS, IGR-N-835), as well for GM-CFC and CFU-F. Regression analysis was used in order to fit the proportion of surviving cells as a function of drug concentration in the IGR-N-835-bone marrow cell mixture, GM-CFC and CFU-F. We applied two types of dose–response models, a linear and an exponential one.

### Results

$IC_{50}$ and $IC_{90}$ values from the three NB cell lines (SK-N-SH, SK-N-AS and IGR-N-835) cultured in a CMA assay were graphically obtained. Values were found to be similar and ranged from 0.4 to 0.8 μM for $IC_{50}$ and from 1 to 7.5 μM for $IC_{90}$ (Table I).

Figure 1 shows the number of remaining colonies of IGR-N-835 cells cultured with normal human bone marrow cells as a function of the CDDP concentration. CDDP produced a total tumoricidal effect at 10 μM. The fit of the data observed with the exponential dose-response model was very satisfactory and the linear model was obviously inadequate. The $IC_{50}$ and $IC_{90}$ values were equal to 1.29 μM (95% CI: 1.14–1.47 μM) and 4.2 μM (95% CI: 3.5–4.9 μM) respectively, as shown in Table I. Given the results obtained concerning NB cells in the mixture, we tried to fit those obtained for GM-CFC and CFU-F cells (Figures 2 and 3), in a range of a concentration below 10 μM which only covers the first decrease in survival of these cells. For both progenitor cells, the $IC_{50}$ and $IC_{90}$ value estimations obtained with the linear and the exponential dose–response models were similar (Table I). Our estimations can be defined as an average of the values of the two models: the $IC_{50}$ value was 70 μM for GM-CFC and 367 μM for CFU-F while the $IC_{90}$ value was 157.5 μM for the GM-CFC and 906.5 μM for the CFU-F. Table II indicates the estimated remaining surviving cell proportions of GM-CFC and CFU-F in the mixture, as a function of the CDDP concentration. In Table II lower and higher values of the 95% confidence intervals of the estimated remaining proportion of cells are based, respectively, on the lower and the higher 95% confidence values obtained in the Table I.

**Table I** $IC_{50}$ and $IC_{90}$ values of various human cell lines and bone marrow cells exposed CDDP as measured in a clonogenic assay

| *Cell lines* | *$IC_{50}$ (μM)* | *95% CI (μM)* | *$IC_{90}$ (μM)* | *95% CI (μM)* |
|---|---|---|---|---|
| Neuroblastoma | | | | |
| IGR-N-835 | 0.4 | (a) | 1 | (a) |
| SK-N-AS | 0.7 | (a) | 5 | (a) |
| SK-N-SH | 0.8 | (a) | 7.5 | (a) |
| Mixed culture | | | | |
| IGR-N-835 | 1.29 | 1.14–1.47 | 4.1 | 3.5–4.9 |
| Bone marrow | | | | |
| GM-CFC | | | | |
| Linear model | 85 | 75–99 | 156 | 142–173 |
| Exponential model | 54 | 49–59 | 159 | 145–145 |
| CFU-F | | | | |
| Linear model | 383 | 352–419 | 707 | 652–952 |
| Exponential model | 351 | 319–392 | 1106 | 1000–1237 |

[a]Graphic estimation

**Figure 1** Remaining number of IGR-N-835 colonies, cultured at a ratio of 10% with normal human bone marrow cells, after 1 h of treatment with various concentrations of CDDP.

**Figure 3** Survival proportion of CFU-F colonies after 1 h of treatment with various concentrations of CDDP.

**Figure 2** Survival proportion of GM-CFC colonies after 1 h of treatment with various concentrations of CDDP.

From these results, two therapeutic indices have been estimated as the ratio of $IC_{50}$ or $IC_{90}$ values between normal haematopoietic progenitor cells and tumour cells (Table I). Given $IC_{50}$ and $IC_{90}$, the therapeutic indices have been found to be equal to about 50 and 40, respectively.

### Discussion

Our results show that CDDP could be an efficient purging agent for NB in ABMT purging conditions. According to the exponential model, a concentration of 39 μM, i.e. 30-fold $IC_{90}$ (95% CI) should reduce the number of tumour cells in a mixture (IGR-N-835 and bone marrow cells) by $10^9$.

Cytological involvement of less than 0.1% may exist in bone marrow selected for ABMT but remains undetectable (Bayle *et al.*, 1985). However, in our mixture, we decided on a ratio of 10% of NB cells so that optimal conditions were achieved with a sufficient number of cell colonies. Tumour cell survival was estimated using a clonogenic assay to explore the possible long-term proliferative capacity of NB cells. In order to evaluate the differential susceptibility of tumour cells according to their concentration in bone marrow mixtures, we carried out another set of experiments using mixtures containing 1% of NB cells and similar results were obtained (data not shown).

With regard to GM-CFC, three studies have been published. Ogawa *et al.* (1975) pointed out that human cells were less sensitive to CDDP than murine GM-CFC. The other two papers studied human tumour cells and human GM-CFC in the presense of CDDP, but separately (Ajani *et al.* 1985; Umbach *et al.*, 1985). None of these studies was performed in ABMT routine purging conditions.

High-dose chemotherapy is currently used to eradicate residual disease in NB patients in complete remission (Hartmann *et al.*, 1985). Asta-Z, an active metabolite of cyclophosphamide at 100 μM, is currently used in NB bone marrow purging. Asta-Z treated marrow, however, induces a

**Table II** Therapeutic index scale of cisplatinum

| Dose | Remaining survival cell proportion (p) of | | | | | |
|---|---|---|---|---|---|---|
| | *IGR-N-835* | | *GM-CFC* | | *CFU-F* | |
| *(μM)* | *p* | *(95% CI)* | *p* | *(95% CI)* | *p* | *(95% CI)* |
| 15 | $3 \times 10^{-4}$ | $(10^{-4}, 8 \times 10^{-4})$ | 0.86 | (0.80–0.89) | 0.97 | (0.96–0.98) |
| 20 | $2 \times 10^{-5}$ | $(5 \times 10^{-6}, 8 \times 10^{-5})$ | 0.82 | (0.75–0.87) | 0.96 | (0.95–0.97) |
| 25 | $10^{-6}$ | $(3 \times 10^{-7}, 8 \times 10^{-6})$ | 0.78 | (0.70–0.84) | 0.95 | (0.94–0.96) |
| 30 | $10^{-7}$ | $(10^{-8}, 7 \times 10^{-7})$ | 0.74 | (0.65–0.81) | 0.94 | (0.93–0.95) |
| 35 | $7 \times 10^{-9}$ | $(6 \times 10^{-10}, 7 \times 10^{-8})$ | 0.71 | (0.61–0.78) | 0.93 | (0.92–0.94) |
| 40 | $5 \times 10^{-10}$ | $(3 \times 10^{-11}, 6 \times 10^{-9})$ | 0.67 | (0.57–0.76) | 0.92 | (0.91–0.93) |

more prolonged delay in recovery from aplasia; almost 4 weeks versus 2.5 without bone marrow purge (Beaujean et al., 1987). In order to reconstitute the haematopoietic system after purge in vitro, $10^8$ bone marrow cells per kilogram are reinjected to the patients. If previously harvested bone marrow is contaminated, ABMT procedure cannot be applicable. Maximal proportion of cytologically measureable occult tumour cells in the infused bone marrow sample is less than 0.1%. This means that, for a patient of 70 kg, about $10^{10}$ bone marrow cells and, possibly, $10^7$ tumour cells are reinjected. Our results show that a dose of 39 μM of CDDP is an adequate purging dose for $10^7$ tumour cells (Table II) while more than 70% of the GM-CFC and more than 90% of the CFU-F cells remain alive at this dose.

Furthermore, CDDP has been shown to induce cell differentiation (Tonini et al., 1986). Whether this agent is able to induce similar effects on NB cells remains to be elucidated. If CDDP-induced cell differentiation were to be established then this, associated with the apparently direct cytotoxic effect of this agent would provide an extremely powerful means for ridding the marrow of metastatic cells with minimal damage to the bone marrow. It would also be of interest to investigate CDDP toxicity to pluripotential haematopoeitic cells.

We are indebted to Lorna Saint Ange for reviewing the manuscript and to Jean Lemerle (Département de Pédiatrie, Institut Gustave Roussy) for his interest in this work. Sterile water for media preparation was Volvic water, generously provided by Volvic Co. (Puy-de-Dôme, France). This work was supported by grants from Institut Gustave Roussy (Contrat de Recherche Clinique 84D8).

### References

AJANI, J.A., BLAAUW, A.A., SPITZER, G. and 6 others (1985). Differential cytotoxic activity of chemotherapy agents on colony-forming cells from human tumors and normal bone marrow *in vitro*. *Exp. Hematol.*, **13**, suppl. 16, 95.

AJANI, J.A. SPITZER, G., TOMASOVIC, B. and 3 others (1986). *In vitro* cytotoxicity patterns of standard and investigational agents on human bone marrow granulocyte-macrophage progenitor cells. *Br. J. Cancer*, **54**, 607.

BAYLE, C., ALLARD, T., RODARY, C. and 3 others (1985). Detection of bone marrow involvement in neuroblastoma: comparison of two cytological methods. *Eur. Pediatr. Haematol. Oncol.*, **2**, 123.

BEAUJEAN, F., HARTMANN, O., PICO, J.L. and 4 others (1987). Incubation of autologous bone marrow graft with Asta-Z (7557): comparative studies of haematologic reconstitution after purged or not purged bone marrow transplantation. *Pediatr. Haematol. Oncol.*, **4**, 105.

CARNEY, D.N., BUNN, P.A., GAZDAR, A.F. and 2 others (1981). Selective growth of small cell carcinoma of the lung obtained from patient biopsies in serum free hormone supplemented medium. *Pro. Natl Acad. Sci. USA*, **78**, 3185.

HARTMANN, O., KALIFA, C., BEAUJEAN, F. and 3 others (1985). Treatment of advanced neuroblastoma with two consecutive high-dose chemotherapy regimens and ABMT. In *Advances in Neuroblastoma Research*, Evans, A.E. (ed.) p. 565. Alan R. Liss: New York.

KAHN, E., BENARD, J. & DI PAOLA, R. (1986). The use of an image analyser in human tumour clonogenic assays. *Cytometry*, **7**, 313.

LAPORTE, J. PH., GORIN, N.C., DOUAY, L. and 13 others (1987). Autogreffe de moelle osseuse traitée par chimiothérapie *in vitro* (Asta Z 7557) en consolidation des leucémies aigües de l'adulte en première rémission complète. *Presse Méd.*, **7**, 338.

OGAWA, M. GALE, G.R. & KEIRN, S.S (1975). Effect of *cis*-diamminedichloroplatinum (NSC 119875) on murine and human hemopoietic precursor cells. *Cancer Res.*, **35**, 1398.

RASO, V., RITZ, J., BASALA, M. and 1 other (1982). Monoclonal antibody-ricin-A chain conjugate selectively cytotoxic for cells bearing the common acute lymphoblastic leukemia antigen. *Cancer Res.*, **42**, 457.

REISNER, Y. & GAN, J. (1985). Differential binding of soybean agglutinin to human neuroblastoma cell lines: potential application to autologous bone marrow transplantation. *Cancer Res.*, **45**, 4026.

SAARINEN, U.M., COCCIA, P.F., GERSON, S.L. and 2 others (1985). Eradication of neuroblastoma cells *in vitro* by monoclonal antibody and human complement: method for purging autologous bone marrow. *Cancer Res.*, **45**, 59.

SHARKIS, S.J., SANTOS, Q.W. & COLVIN, M. (1980). Elimination of acute myelogenous leukemic cells from marrow and tumor suspension in the rat with 4-hydroperoxycyclophasphamide. *Blood*, **55**, 521.

SIENA, S., GASTRO-MALASPINA, H., GULATI, S.C. and 5 others (1985). Effects of *in vitro* purging 4-hydroperoxycyclophosphamide on the hematopoietic and microenvironmental elements of human bone marrow. *Blood*, **65**, 655.

TONINI, G.P., PARODI, M.T., BOLOGNA, R. and 3 others (1986). Cis-platin induces modulation of transferrin receptor during cellular differentiation *in vitro*. *Cancer Chemother. Pharmacol.*, **18**, 92.

TREALEAVEN, J.G., GIBSON, F.M., UGELSTAD, J. and 4 others (1984). Removal of neuroblastoma cells from bone marrow with monoclonal antibodies conjugated to magnetic microspheres. *Lancet*, **i**, 70.

UMBACH, G.E., SINGLETARY, S.E., TOMASOVIC, B. and 3 others (1984). Dose survival curves of cis-platinum, melphalan and velban in human granulocyte/macrophage progenitor cells. *Int. J. Cell Cloning*, **2**, 335.

UMBACH, G.E., HUG, V., SPITZER, G. and 4 others (1985). Survival of human bone marrow cells after *in vitro* treatment with 12 anticancer drugs and implications for tumor drug sensitivity assays. *J. Cancer Res. Clin. Oncol.*, **109**, 130.